# Does the phaco TIp position during clear corneal Phacoemulsification Surgery adversely affect corneal endothelium? TIPS study protocol for a randomised, triple-masked, parallel-group trial of bevel-up versus bevel-down phacoemulsification

**DOI:** 10.12688/wellcomeopenres.16098.1

**Published:** 2020-07-16

**Authors:** Soujanya Kaup, Siddharudha Shivalli, Chinnappa Ajjinicanda Ganapathi, Cynthia Arunachalam, John Buchan, Suresh Kumar Pandey, Krishna Prasad Kudlu

**Affiliations:** 1 Department of Ophthalmology, Yenepoya Medical College Hospital, Yenepoya Deemed to be University, Mangalore, Karnataka, 575018, India; 2 Department of Medical Statistics, London School of Hygiene and Tropical Medicine, Keppel Street, London, WC1E 7HT, UK; 3 Department of Ophthalmology, Netra Jyothi Charitable Trust Hospital, Udupi, Karnataka, 576101, India; 4 International Centre for Eye Health, London School of Hygiene and Tropical Medicine, Keppel Street, London, WC1E 7HT, UK; 5 Department of Ophthalmology, SuVi Eye Institute and Lasik Laser Center, Kota, Rajasthan, 324005, India

**Keywords:** Endothelial cell loss, phacoemulsification, phacoemulsification/complication, pseudophakic bullous keratopathy, Phaco-tip, bevel-up, bevel-down, specular microscopy

## Abstract

**Introduction:** Globally, at least 30 million cataract surgeries are required annually to prevent cataract-related blindness. Corneal endothelial decompensation is one of the most common causes of poor visual outcome following cataract surgery, particularly in those with predisposing factors. The increasing ageing population and reduced visual impairment threshold for cataract surgery have resulted in rising cataract surgical rates and hence, an increase in corneal endothelial decompensation is expected. The role of phaco tip position on corneal endothelial damage is ambiguous. Previous studies have reported contradictory results and were also underpowered to detect a significant difference due to small sample sizes. With no consensus regarding the most cornea-friendly phaco tip position (bevel-up versus bevel-down) during phacoemulsification, we propose a randomised clinical trial with a robust design using direct chop phaco-technique.

**Objective:** To compare the effect of phaco tip position (bevel-up vs. bevel-down) on corneal endothelial cell count during phacoemulsification.

**Methods:** A randomised, multicentre, parallel-group, triple-masked (participant, outcome assessor, and statistician) trial with 1:1 allocation ratio is proposed. By adopting stratified randomisation (according to cataract grade), we will randomly allocate 480 patients aged >18 years with immature cataract into bevel-up and bevel-down groups at two centres. History of significant ocular trauma, previous intraocular surgery, shallow anterior chamber, low endothelial cell count, pseudoexfoliation syndrome, intraocular inflammation, and corneal endothelial dystrophy are the key exclusion criteria. The primary outcome is postoperative endothelial cell count at one month. Secondary outcomes are central corneal thickness on postoperative days 1, 15, and 30, and intraoperative complications.

**Trial registration:** Clinical Trial Registry of India CTRI/2019/02/017464 (05/02/2019).

## Introduction

Cataract causes blindness or moderate to severe visual impairment in about 62.5 million people globally ^1^. Each year, at least 30 million cataract surgeries are required to prevent cataract-related blindness ^2^. Owing to the increasing burden of cataract (due to the growing ageing population of the world ^3^ and reduced visual impairment threshold for surgery ^4^,) the number of cataract surgeries performed is likely to increase ^2, 5^. Phacoemulsification is the most commonly performed cataract surgery in developed countries and is rapidly increasing in developing countries like India ^6^.

Corneal endothelium pumps fluid out of the corneal stroma, prevents the development of corneal oedema and thus maintains corneal transparency ^7^. Normally, about 0.3–0.6% of the endothelial cells are lost every year ^8, 9^. Corneal endothelial cell loss is likely to increase (in varying amounts) after any intraocular surgery ^10^. Following injury, endothelial cells increase in size and change from a hexagonal to pleomorphic shape ^9^. Persistent corneal oedema can occur if the injury causes significant endothelial cell loss (below the critical density), necessitating corneal transplantation.

Corneal endothelial decompensation is a common cause of post-operative poor vision following cataract surgery with a reported incidence of 0.5–2% of cataract surgeries ^11, 12^. Phacoemulsification, particularly in those with certain predisposing factors, results in significant endothelial cell damage and loss, with corneal decompensation ^13^ and is one of the leading indications for corneal transplant across the globe. Corneal decompensation constitutes 28% of all the keratoplasties in North America; 20.6% in Europe; 21.1% in Australia; 13.6% in the Middle East; 15.5% in Asia, and 18.6% in South America ^14^. Hence, with an increase in the number of cataract surgeries, a significant increase in the incidence of corneal endothelial decompensation is anticipated.

Old age, increased nucleus density and high ultrasound energy increase the risk of endothelial cell loss during phacoemulsification ^13, 15, 16^. To minimise the corneal endothelial cell loss, various modulations in phaco platforms and different phaco-surgical techniques are introduced. The magnitude of endothelial cell loss is directly related to the amount of ultrasound energy used ^16^. Hence, power modulation by various means (e.g. microburst techniques) is a provision with most phaco machines to reduce the amount of ultrasound energy. Additionally, different phaco techniques that decrease the amount of ultrasound energy used are employed ^17– 21^.

Despite advancement in the phacoemulsification technique, corneal endothelial damage continues to be a key concern. The proportion of endothelial cell loss that is accounted for by the choice of phaco tip position is uncertain. It is speculated that the phaco tip, considered to be the source of heat, when kept away from the corneal endothelium with the bevel-up technique might result in minimal cell loss ^22^. However, in this position, the cavitational energy is directed towards the endothelium, which may have a negative impact. It is also possible that the bevel-down technique is more cornea-protective, with better contact between the phaco tip and the nucleus, making power delivery and aspiration more effective ^23, 24^.

Previously published studies investigating the impact of phaco tip position on the endothelium have reported contradictory results ^22, 25, 26^. Moreover, these studies were underpowered to detect a significant difference due to small sample sizes (n= 25 to 30 in each group). In an artificial eye model study, Frohn *et al*. reported that there was no significant difference (n=30 experiments, p= 0.7869) in the amount of ultrasound waves reaching the cornea in bevel-up and bevel-down positions ^27^. However, an artificially controlled environment study might not mirror natural eye conditions.

Joshi *et al.* compared different phaco parameters of ‘phacoemulsification with a 0-degree phaco tip’ and ‘30-degree phaco tip with combination of bevel-up and bevel-down phacoemulsification’, and found no significant difference in both groups. However, they did not compare the effect of these manoeuvres on corneal endothelial cell loss. Hence, there is no consensus regarding the most cornea friendly phaco tip position during phacoemulsification ^28^.

The authors were previously conducting a clinical trial exploring the effect of phaco tip position on central corneal thickness (CCT) during phacoemulsification ^29^. CCT was the primary outcome as no specular microscope was available. CCT is not a definitive measure of corneal endothelial cell loss, as it is affected by other factors such as glucose and HbA1c levels. CCT is also known to display diurnal variation; being thickest in the morning and gradually thinning as the day progresses ^30– 33^.

To answer this long-standing clinical question, we propose a randomised clinical trial with a robust study design using direct chop phacoemulsification technique and specular microscopy, which can non-invasively analyse the morphology of endothelial cells.

### Objective

To compare the effect of phaco tip position (bevel-up vs. bevel-down) on corneal endothelial cell count during phacoemulsification.

### Trial design and registration

Randomised, multicentre, parallel-group, triple-masked (participant, outcome assessor, and statistician) trial with 1:1 allocation ratio. The trial is prospectively registered in the Clinical Trial Registry of India ( CTRI/2019/02/017464; registered on 05/02/2019) with all items from the World Health Organization Trial Registration Data Set. This is trial protocol version 3 (15/09/2018); the previous two versions have not been published.

## Methods

### Ethical statement

The study protocol was approved by the ethics committees of Yenepoya (Deemed to be) University, Mangalore, India [YEC-1/217/2019] and Manipal Academy of Higher Education, Manipal, India [MAHE/ EC/05-19/06]. Any modifications in the trial protocol would require ethics committee approval and the same shall be communicated to Data Monitoring Committee and Clinical Trial Registry of India. The study will comply with the Declaration of Helsinki guidelines, local laws, and the International Council for Harmonisation - Good Clinical Practice (ICH-GCP) guidelines. After obtaining written informed consent from all study participants, the investigators will replace participant identifiers with unique research codes. Investigators will restrict access to research data by keeping the completed case report forms in a locked room and by using password-protected electronic files. All the research participants are insured, and any trial-related complications will be compensated for. Participants will be reimbursed for their travel expenses for follow-up visits.

### Study settings

1. Department of Ophthalmology, Yenepoya Medical College Hospital, Yenepoya (Deemed to be) University, Mangalore, India.
2. Netrajyothi Charitable Trust Hospital, Udupi, India

### Study period

September 2018 to September 2023.

### Inclusion criteria

Patients aged >18 years with immature cataract attending the two study centres in Karnataka, India.

### Exclusion criteria

History of significant ocular trauma, previous intraocular surgery, shallow anterior chamber (<2.5 mm), endothelial cell count <1500 cells/mm ^2^, pseudoexfoliation syndrome, previous/current intraocular inflammation (cells/flare/pigment over an anterior capsule or endothelium/posterior synechiae), preoperative fully dilated pupil <5 mm, and/or corneal endothelial dystrophy (presence of corneal guttae noted on slit lamp examination or specular microscope), and patients on oral tamsulosin or doxazosin. Cases with complications (posterior capsular rent, vitreous loss, zonular dialysis, nucleus drop, suprachoroidal haemorrhage, Descemet’s membrane stripping intraoperatively, and postoperative endophthalmitis) will also be excluded from the analysis, but the rates of any post-randomisation exclusion events will be recorded and reported for per protocol analysis. Intention-to-treat analysis will also be done without any post-randomisation exclusions. However, complications that occur before the intervention, leading to conversion to manual small incision cataract surgery, will be excluded from the analysis.

### Randomisation and masking

**Figure 1.**
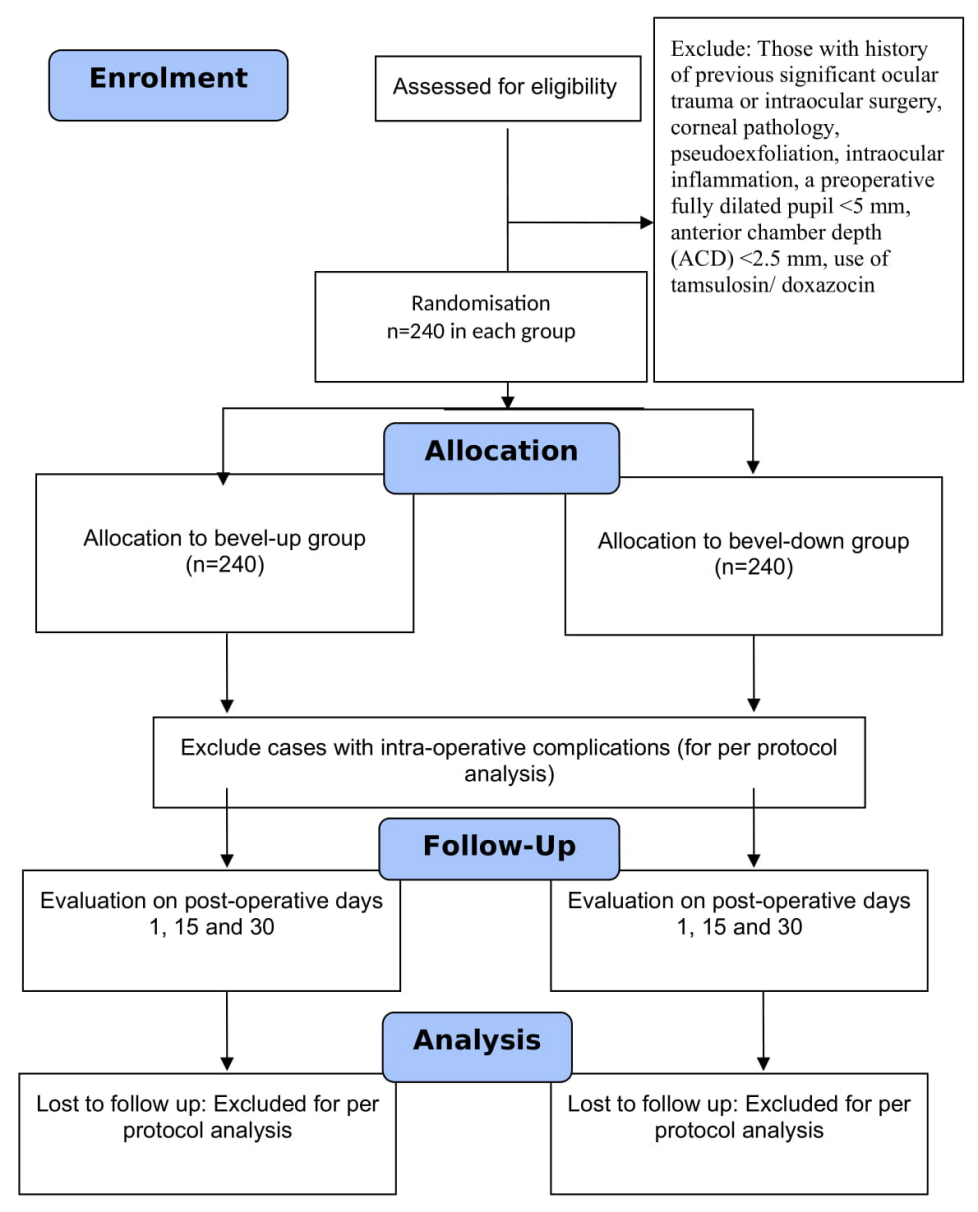
Proposed flow of participants in this trial.

SK/CAG will approach potentially eligible participants attending the outpatient departments of the study hospitals. A research assistant at the study site will provide detailed information about the trial and obtain written informed consent. SK/ CAG will enrol the consenting participants after screening for exclusion criteria. SS will generate a random number sequence using a computer, which will be stored in secured envelopes. Central randomisation with stratified blocks of variable size will be used. Stratification will be done according to the Lens Opacities Classification System (LOCS) III grading of the cataract into two strata (Strata 1: Grade 1, 2 and Strata 2: Grade 3, 4) ^34^. On the day of surgery, SK/CAG will contact the central randomisation unit and SS will allocate the participants into either of the two groups, i.e., bevel-up or bevel-down ( Figure 1). SS will not be in direct contact with the participants. The trial participant, outcome assessor, and statistician will be masked. The participant will not be aware of the group to which they were randomised and will not be able to differentiate the interventions. The outcome will be assessed by a trained research assistant who is unaware of the intervention. An independent statistician, who is unaware of the random allocation, will analyse the data.

### Interventions

**Figure 2.**
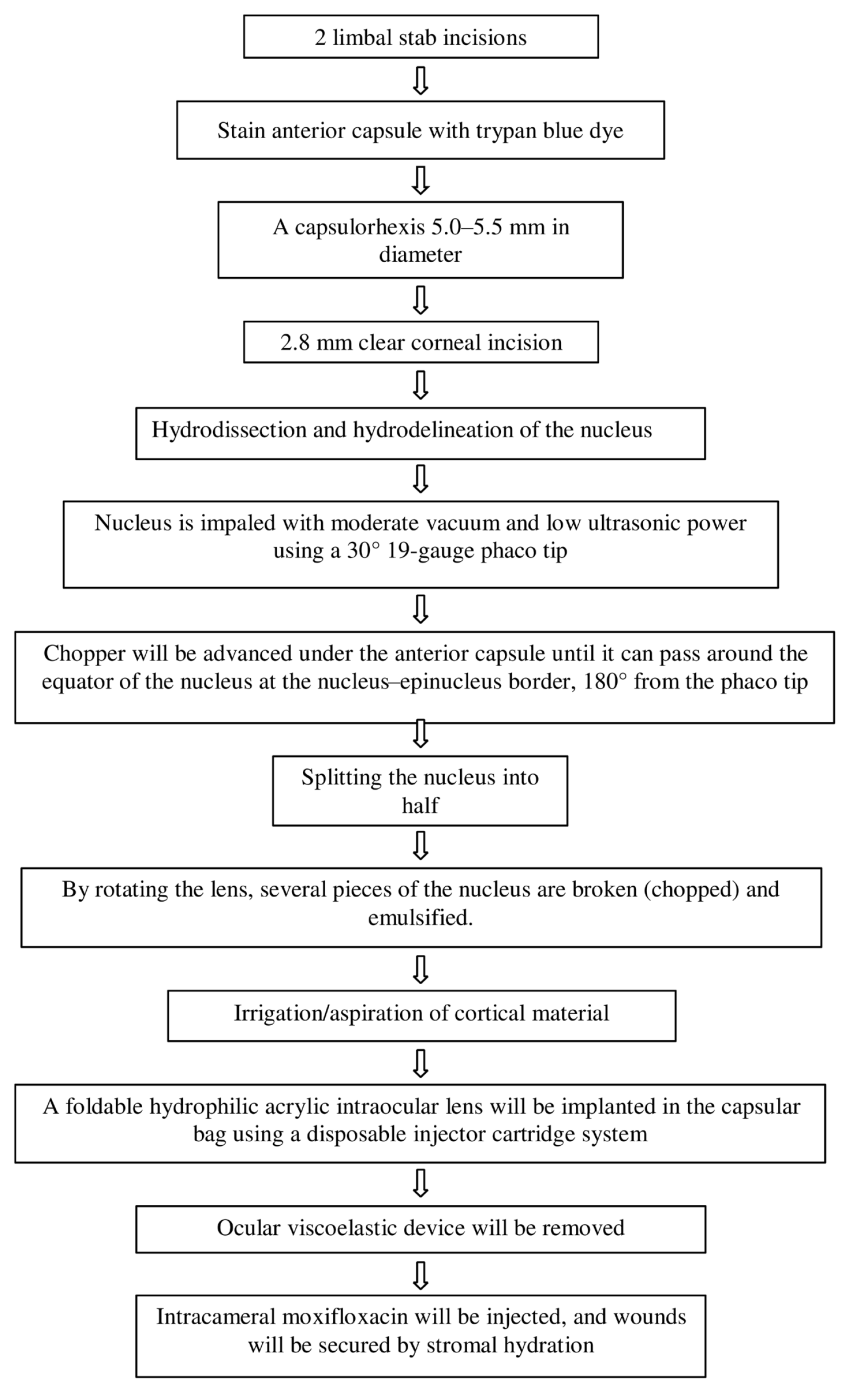
Surgical procedure.

Figure 2 shows the steps of the surgery. All surgeries will be performed under the peribulbar block. The bevel of the phaco tip will be held facing up during nucleus management in patients randomised to the “bevel-up group” and down in the “bevel-down group”. Balanced salt solution (BSS; Intasol, Intas pharma) with 1:1000 adrenaline (0.5 ml in 500 ml of BSS) (Epitrate, Sunways) will be used. Intracameral lignocaine, phenylephrine, or pilocarpine will not be used.

All surgeries will be performed by a single ophthalmic surgeon at each study site (SK and CAG). To familiarise surgeons with the techniques, each surgeon will perform at least 25 surgeries using each technique before the start of the trial. No strategies to improve adherence to intervention is required as it is a one-time procedure.

### Phaco platform and parameters

We will use the Sovereign compact phacoemulsification system with WhiteStar technology and Ellips (Abbott Medical Optics, Abbott Laboratories, Abbott Park, Illinois, USA) for all the surgeries. Following are the parameters for the direct chop: maximum aspiration flow rate: 32 cc/min; maximum vacuum: 300 to 410 mm Hg; threshold vacuum: 170 mm Hg; and maximum power: 40 linear long pulse 8/12 (40%) with Whitestar on and occluded: 40 linear short pulse 6/12 (33%) with Whitestar on. A 19-gauge, 30-degree phaco tip will be used for all surgeries.

### Preoperative evaluation

Preoperative evaluation includes uncorrected and corrected distance visual acuity (UDVA and CDVA), slit-lamp examination, applanation tonometry, an examination of retina with a non-contact 78 dioptre lens, and indirect ophthalmoscopy. Maximum pupillary dilatation will be noted 20 minutes after instillation of tropicamide with phenylephrine eye drops. The axial length (AL) and anterior chamber depth will be measured using an ultrasonic A-scan (Echorule Pro, Biomedix Optotechnik & Devices, Bangalore, India) or optical biometer (IOL Master 500, Carl Zeiss). CCT will be measured using an ultrasound pachymeter (Pacscan 300P, Ver 3 Rev U, Sonomed Escalon, Lake Success, NY) with an SD ≤0.09, with the patient fixating on a distant target. Endothelial cell density will be measured using specular microscope SP-1P (Topcon Europe Medical BV, Netherlands). Based on the nucleus colour, we will clinically estimate the hardness and grade according to LOCS III ^34^.

### Intraoperative evaluation

We will note the mean phaco power (%), ultrasound time (UST), effective phaco time (EPT) (seconds) and the amount of irrigating fluid used.

### Postoperative treatment

A combination of topical moxifloxacin and dexamethasone eye drops, one drop six times a day for the first week and gradually tapered over one month, will be administered.

### Postoperative evaluation

The following examinations will be done on day 1, day 15 and at the end of one month: UDVA, CDVA, slit lamp biomicroscopy, applanation tonometry, fundoscopy and CCT measurement. The endothelial count will be assessed at the end of one month. The coefficient of variation of cell size and percentage of hexagonal cells will also be measured. Automatic focusing and digital image capture will be used. In the case of a blurred and noisy image, the cells will be identified manually. The approximate centre of the cell will be marked using a stylus pen on the captured specular digital image. The guidelines for the use of the specular microscope in clinical trials as proposed by McCarey *et al.* will be followed ^35^. Endothelial cell evaluation will be done through the same specular microscope throughout the study period at each site. Regular follow-up visits of the patients will be encouraged through telephonic reminders.

### Outcome measures

Primary outcome: Endothelial cell count at one month postoperatively.

Secondary outcome: CCT on days 1, 15, and 30. Intraoperative complications will also be noted.

### Sample size calculation

Based on a pooled standard deviation of 441.7, this study would require a sample size of 215 for each group to achieve a power of 90% and a level of significance of 5% (two-sided), for detecting a true difference of 138 cells/mm ^2^ (2516 - 2378) in the means between the study groups ^26, 36^. Expecting 10% attrition in this trial, we would recruit 240 eligible participants in each group (total 480).

### Data collection and statistical analysis

Research assistants will collect all the relevant data on a case report form (CRF). Research assistants at each study site will independently enter the data from CRF into a password protected server. SK will regularly perform source data verification. We will follow double data entry method to identify data entry errors. In case of any discrepancy, the data query would be sent to the research assistant at the trial site to re-check the source data and inform the changes, if any. Any changes made in the CRF will be signed and dated to have an audit track.

A blind review of the data will be performed. The analysis will follow the intention-to-treat principle. ‘Per protocol’ analysis will also be performed excluding patients who experience intraoperative and postoperative complications, as the complications themselves can have a direct impact on the endothelial count. Descriptive statistics will be used to express the results. We will compare the mean endothelial cell counts between the study groups by bi-variate analysis. To assess the CCT difference between the study groups, repeated measures analysis of variance will be used. Statistical Package for Social Sciences (SPSS) version 16.0 (SPSS Inc. Chicago, USA) will be used.

### Data monitoring

A data monitoring committee (DMC) with independent members is constituted (see *Extended data*) ^37^. Based on their findings, the DMC will recommend continuation, modification or discontinuation, of the trial, with reports to the ethics committees.

Investigators will report any serious adverse events to the DMC and ethics committee within 24 hours. We will also enlist all the adverse events and report using descriptive statistics. We will compare the adverse events between the study groups.

### Dissemination

The authors will present the results of the trial in conferences and publish them in relevant journals. All the de-identified data will be uploaded in an online repository at the end of the trial.

### Study status

The trial is in the recruitment phase.

## Discussion

Corneal endothelial cells are precious as they do not regenerate, and they only decrease with age. Although endothelial damage of varying degrees is known to occur in all intraocular procedures, techniques that minimise the endothelial damage should be favoured. Hence, ophthalmologists are continually striving to find a more cornea friendly technique of cataract surgery. Endothelial cell loss is of utmost importance in corneas predisposed to bullous keratopathy (such as those with Fuchs endothelial dystrophy) or in eyes likely to have more serious endothelial cell damage (e.g., those with a hard nucleus, old age, small pupil, and shallow anterior chamber) ^13, 16, 38^.

The quantum of corneal endothelial loss during phacoemulsification seems to be mainly determined by the heat generated at the phaco tip, cavitation energy, and the amount of ultrasound used ^22– 24^. The phaco tip position is likely to determine the impact of these factors on the corneal endothelium. Hence, it would be worthwhile exploring the phaco tip position (bevel-up or bevel-down) during phacoemulsification resulting in minimum corneal endothelial cell loss.

In a trial of 60 patients by Faramarzi *et al.*, the mean (SD) corneal endothelial cell loss was significantly lower (p=0.017) in the bevel-up group (156 ± 150) when compared to the bevel-down group (332 ± 363). On the contrary, Raskin *at el.* (n=25 in each group) reported that postoperative mean (SD) endothelial cell count was significantly more (p=0.02) in the bevel-down (2252 ± 310) when compared to bevel-up group (2393 ± 321). Based on the sample size and actual observed difference between the study groups, the powers of the studies were 69% and 36% for Faramarzi *et al.* and Raskin *et al.*, respectively ^39^. Hence, neither of these studies had enough patients to detect whether a significant difference truly exists between the study groups. Moreover, potential confounders such as cataract grade and masking were not explicitly addressed during randomisation or analysis.

A clinical trial can give rise to erroneous results through the introduction of bias/systematic errors, confounding (which can be restricted by randomisation) and random error (which can be minimised by using a large sample size) ^40^. Keeping in view the limitations of the previous trials ^22, 25, 26^, we have adopted a robust design (stratified randomisation based on the cataract grade and triple-masking) with adequate sample size to detect the expected difference in the endothelial cell loss between the two groups. Additionally, this trial utilises specular microscopy, which is a fairly objective and non-invasive method of measuring the corneal endothelial cell count and morphology ^41^.

## Conclusion

The proposed trial results will guide ophthalmic surgeons in choosing the most cornea friendly phaco tip position during phacoemulsification and subsequently minimise the incidence of iatrogenic bullous keratopathy.

## Data availability

### Underlying data

No underlying data are associated with this article.

### Extended data

Open Science Framework: Does the phaco-TIp position during clear corneal Phacoemulsification Surgery adversely affect corneal endothelium? TIPS study protocol for a randomised, triple-masked, parallel-group trial of bevel-up versus bevel-down phacoemulsification. https://doi.org/10.17605/OSF.IO/5YS6W ^37^

This project contains the following extended data:

- DMC charter.docx (Data Monitoring Committee charter)
- Informed consent form.docx (informed consent form in English)
- Netra jothi kannada consent 27.5.2019.pdf (informed consent form in Kannada)
- Malayalum consent 27.5.2019.pdf (informed consent form in Malayalum)

### Reporting guidelines

Open Science Framework: SPIRIT and TIDieR checklists for “Does the phaco-TIp position during clear corneal Phacoemulsification Surgery adversely affect corneal endothelium? TIPS study protocol for a randomised, triple-masked, parallel-group trial of bevel-up versus bevel-down phacoemulsification” https://doi.org/10.17605/OSF.IO/5YS6W ^37^

Data are available under the terms of the Creative Commons Zero "No rights reserved" data waiver (CC0 1.0 Public domain dedication).
