# Supplementary material for: Does the phaco TIp position during clear corneal Phacoemulsification Surgery adversely affect corneal endothelium? TIPS study protocol for a randomised, triple-masked, parallel-group trial of bevel-up versus bevel-down phacoemulsification
Source: Wellcome Open Res. 2023 Jan 25;5:167. Originally published 2020 Jul 16. [Version 2] doi: 10.12688/wellcomeopenres.16098.2 (PMC10767251; doi:10.12688/wellcomeopenres.16098.2)
Supplement: Peer review history

# Reviewer report: Reviewer response for version 2

**DOI:** 10.21956/wellcomeopenres.20845.r69451

**Published:** 2024-01-04

**Authors:** Sharon D'Souza

**Affiliations:** 1 Department of Cornea and Refractive surgery, Narayana Nethralaya, Bengaluru, Karnataka, India

The study has addresses an important knowledge gap in our understanding of phacoemulsification surgery. 

 Even though this question has been studied before, the larger study cohort, comparison groups and robust protocol will add to the scientific knowledge regarding this topic.

 The authors have done a good review of literature for relevant articles on this topic. The objective is clear and simple and the study design has been well formulated around this.  

 One of the exclusion criteria is an anterior chamber depth of < 2.5mm. The authors should definitely look at anterior chamber depth and ensure not too much variation even among the normal. 

 Other features of endothelial cells on specular microscopy like pleomorphism, CV value and hexagonality should also be noted as endothelial cell density would not be the only parameter affected.

 AS different surgeons have variations in technique, the number of surgeons is a confounding factor.

 It is good that the phacoemulsification parameters are fixed across surgeons and machines. 

 The inclusion and exclusion criteria are adequate.

Is the study design appropriate for the research question?

Yes

Is the rationale for, and objectives of, the study clearly described?

Yes

Are sufficient details of the methods provided to allow replication by others?

Yes

Are the datasets clearly presented in a useable and accessible format?

Yes

Reviewer Expertise:

Cornea, ocular surface and ocular immunology

I confirm that I have read this submission and believe that I have an appropriate level of expertise to confirm that it is of an acceptable scientific standard.

# Reviewer report: Reviewer response for version 2

**DOI:** 10.21956/wellcomeopenres.20845.r54509

**Published:** 2023-02-27

**Authors:** Anthony Vipin Das

**Affiliations:** 1 Department of EyeSmart EMR & AEye, Kallam Anji Reddy Campus, LV Prasad Eye Institute, Hyderabad, Telangana, India

Thank you for addressing the comments provided in the earlier review.

 I have no further comments on this version.

Is the study design appropriate for the research question?

Yes

Is the rationale for, and objectives of, the study clearly described?

Yes

Are sufficient details of the methods provided to allow replication by others?

Yes

Are the datasets clearly presented in a useable and accessible format?

Yes

Reviewer Expertise:

Cataract Surgery, Big Data, Ophthalmology

I confirm that I have read this submission and believe that I have an appropriate level of expertise to confirm that it is of an acceptable scientific standard.

# Reviewer report: Reviewer response for version 1

**DOI:** 10.21956/wellcomeopenres.17667.r50887

**Published:** 2022-06-21

**Authors:** Anthony Vipin Das

**Affiliations:** 1 Department of EyeSmart EMR & AEye, Kallam Anji Reddy Campus, LV Prasad Eye Institute, Hyderabad, Telangana, India

Thank you for the opportunity to review this study protocol. Please find my observations below.

1. The authors present a robust study design to determine the ideal position of the phaco tip during phacoemulsification. The following two factors must be taken into consideration which are unique for each eye on the table.
2. While the ACD is being determined during the AScan, the actual ACD during the Phacoemulsification is dynamic based on the fluid dynamics in the Anterior Chamber. It is quite high in High Myopes and can be low in patients with occludable angles. In spite of this, there is variability in the ACD that can occur even in seemingly normal eyes. This is a judgement of the operating surgeon and must be considered as the space that is available for the phaco to be performed will vary accordingly. 
3. The other aspect of the distance of the phaco probe from the endothelium is dependent on the plane of the probe during the procedure. The surgeon, if possible, must always maintain it at a constant depth, notably at the plane of the Iris. This will ensure that during the Quad mode of the Phaco Machine, there is a constant plane that is achieved to emulsify the fragments in the anterior chamber.
4. There is still a possibility for the probe to changes its plane as it aspirates the nuclear fragments, but the surgeon variance should be taken care of by two surgeons who will ensure that the major step of quadrant removal through the application of the phaco is performed in a standard plane of the iris, which is away from the endothelium.
5. Another aspect that must be taken into consideration is the LOCS grading of the cataract. While the nuclear sclerosis is determined pre-operatively. The amount of energy that might be required for aspirating the epinucleus and cortex must be judged by the operating surgeon. This is unique to each case and surgeon style on how much of aspiration is used by the probe and when the phaco is applied for the fragments that are harder.
6. It would also help to decide the standard number of fragments that the surgeon is planning to divide the nucleus into. Is it 4 or 6 or sometimes the phaco can be performed in just the 2 fragments in direct chop if they are soft enough and prolapse into the AC. This might help reduce the variance in the surgeon operating style. 
7. The authors might also want to consider one of the most important factor for the health of the endothelium which is the irrigating solution. In reality, the use of BSS is expensive and not wide spread. They must also factor this in the study of the more commoner irrigating solutions used in the field by various surgeons in different ophthalmic practices.
8. The viscoelastic coating of the endothelium before the start of the phacoemulsification is also an important step in protecting the endothelium from the heat generated in the anterior chamber. Is this effect going to be also factored in on the type of viscoelastic used and also the quality of the product. If so, this step must be mentioned in the protocol whether it is being performed.
9. The corneal edema that ensues post the cataract surgery must also be factored in on first post-operative day. This also will indicate the insult caused to the endothelium during the surgery. There must be a standard way to grade the corneal edema on POP Day 1 and then follow up on subsequent visits of resolution of the same. This will also guide on the time taken to resolution of the corneal edema and correlate with the final endothelial count at one month.
10. To engage the fragment, in certain instances a side bevel is used to engage the piece in a more predictable manner. Once the piece is engaged and away from the PC and endothelium, the phaco is applied to emulsify. In such instances, it would be interesting to understand what position the probe will be in while the quad step is being performed.

Please reflect on the above points which might add value to the study design and can incorporate accordingly. Thank you.

Is the study design appropriate for the research question?

Yes

Is the rationale for, and objectives of, the study clearly described?

Yes

Are sufficient details of the methods provided to allow replication by others?

Yes

Are the datasets clearly presented in a useable and accessible format?

Yes

Reviewer Expertise:

Cataract Surgery, Big Data, Ophthalmology

I confirm that I have read this submission and believe that I have an appropriate level of expertise to confirm that it is of an acceptable scientific standard, however I have significant reservations, as outlined above.

# Response

**Published:** 2022-12-26

**Authors:** Soujanya Kaup

**Affiliations:** Yenepoya (Deemed to be) University, India

2. While the ACD is being determined during the AScan, the actual ACD during the Phacoemulsification is dynamic based on the fluid dynamics in the Anterior Chamber. It is quite high in High Myopes and can be low in patients with occludable angles. In spite of this, there is variability in the ACD that can occur even in seemingly normal eyes. This is a judgement of the operating surgeon and must be considered as the space that is available for the phaco to be performed will vary accordingly.

**Response: **We agree with your comments. We cannot quantify the intra-operative ACD variations. We presume that this variation will have occurred randomly in both the trial arms in a similar fashion. There are numerous factors that can affect EC loss during phacoemulsification. We are only trying to see the isolated effect of changing the tip position on EC loss. We are presuming that stratified randomisation (with variable block size) will ensure balancing all known and unknown factors equally among both the trial arms. We have measured ACD pre-operatively and excluded those cases who have ACD of less than 2.5mm which is a known risk factor for increased EC loss.

 3. The other aspect of the distance of the phaco probe from the endothelium is dependent on the plane of the probe during the procedure. The surgeon, if possible, must always maintain it at a constant depth, notably at the plane of the Iris. This will ensure that during the Quad mode of the Phaco Machine, there is a constant plane that is achieved to emulsify the fragments in the anterior chamber.

**Response:** There is still a possibility for the probe to changes its plane as it aspirates the nuclear fragments, but the surgeon variance should be taken care of by two surgeons who will ensure that the major step of quadrant removal through the application of the phaco is performed in a standard plane of the iris, which is away from the endothelium. All surgeries will be performed in the capsular bag. The surgeons are familiar with the standard surgical protocol and have been trained to follow the procedure as per the standard surgical protocol.

 5. Another aspect that must be taken into consideration is the LOCS grading of the cataract. While the nuclear sclerosis is determined pre-operatively. The amount of energy that might be required for aspirating the epinucleus and cortex must be judged by the operating surgeon. This is unique to each case and surgeon style on how much of aspiration is used by the probe and when the phaco is applied for the fragments that are harder.

**Response: **It is probably reasonable for us to assume that the phaco energy is largely employed in nucleus removal rather than epinucleus. However, we will note the total amount of ultrasound energy used at the end of each surgery and compare this between the trial arms.

 6. It would also help to decide the standard number of fragments that the surgeon is planning to divide the nucleus into. Is it 4 or 6 or sometimes the phaco can be performed in just the 2 fragments in direct chop if they are soft enough and prolapse into the AC. This might help reduce the variance in the surgeon operating style.

**Response: **I guess this could be observed retrospectively perhaps, rather than dictating it prospectively, but ideally it would be the same in each arm. The more energy that comes from the chopper, the less comes from the phaco probe, so it would be of potential significance if the surgeon were chopping up into 6 parts for bevel up, but only 2 parts for bevel down. However, as we will be noting and comparing the ultrasound energy used in both the trial arms we will know if we are using more phaco-energy in one group over the other.  

 7. The authors might also want to consider one of the most important factor for the health of the endothelium which is the irrigating solution. In reality, the use of BSS is expensive and not wide spread. They must also factor this in the study of the more commoner irrigating solutions used in the field by various surgeons in different ophthalmic practices.  

**Response: **For the purpose of standardisation, we have used only one type of irrigating fluid. We are not trying to assess the factors contributing towards EC loss. We are only trying to see the isolated effect of phaco-tip position on EC loss keeping all other known (and possible unknown) factors constant.

 8. The viscoelastic coating of the endothelium before the start of the phacoemulsification is also an important step in protecting the endothelium from the heat generated in the anterior chamber. Is this effect going to be also factored in on the type of viscoelastic used and also the quality of the product. If so, this step must be mentioned in the protocol whether it is being performed.

**Response:** We will be using hydroxy propyl methyl cellulose to coat the endothelium uniformly across the trial arms.

 9. The corneal edema that ensues post the cataract surgery must also be factored in on first post-operative day. This also will indicate the insult caused to the endothelium during the surgery. There must be a standard way to grade the corneal edema on POP Day 1 and then follow up on subsequent visits of resolution of the same. This will also guide on the time taken to resolution of the corneal edema and correlate with the final endothelial count at one month. **Response: **We will be assessing the central corneal thickness with pachymetry that will help us in assessing the corneal oedema post-operatively.

 10. To engage the fragment, in certain instances a side bevel is used to engage the piece in a more predictable manner. Once the piece is engaged and away from the PC and endothelium, the phaco is applied to emulsify. In such instances, it would be interesting to understand what position the probe will be in while the quad step is being performed.

**Response: **For the purpose of the trial, we have standardised the surgical protocol to be followed. For all the surgeries under bevel-up group, the probe will be positioned in bevel-up position throughout the procedure and vice versa for bevel-down group. Even if the surgeon, tilts the probe momentarily to engage the fragment, they will emulsify the fragment only in the assigned position (bevel-up or bevel-down). This will be verifiable from the video excerpts which will be viewed by an external surgeon, masked to allocation, who will confirm which technique was being utilised. This now updated in the protocol.

# Reviewer report: Reviewer response for version 1

**DOI:** 10.21956/wellcomeopenres.17667.r42165

**Published:** 2021-02-01

**Authors:** Peter A Mattei

**Affiliations:** 1 Department of Medicine and Science of Ageing, Ophthalmology Clinic, University G. D'Annunzio Chieti-Pescara, Chieti, Italy

The trial design describes a randomised, multicentre (two centres with on surgeon per centre), parallel-group, triple-masked (participant, outcome assessor, and statistician) trial with 1:1 allocation ratio of 480 patients (240 per phacoemulsification technique: bevel-up vs. bevel-down) to evaluate the endothelial cell count at one month postoperatively (primary outcome) and CCT on days 1, 15, and 30 (secondary outcomes).

 My primary concerns are:

1. Lack of controls to determine if the bevel-up vs. bevel-down technique was followed as per allocation.
2. What percentage (time or energy) of the phacoemulsification must be done with the tip in this position. This aspect will probably affect the sample size calculation, as would the inclusion of confounding parameters such as centre/surgeon and grading strata.
3. The follow-up time would probably not be long enough to see the final endothelial cell loss or decrease in central thickness. see for example DOI: 10.1007/s10792-016-0283-7
4. I was unable to locate the data-set, maybe my fault.
5. How will the endothelial cell loss be recorded, absolute loss or percentage decrease, for the evaluation of between technique differences?
6. SPSS 16 is a bit outdated. Consider using R?

Is the study design appropriate for the research question?

Partly

Is the rationale for, and objectives of, the study clearly described?

Yes

Are sufficient details of the methods provided to allow replication by others?

No

Are the datasets clearly presented in a useable and accessible format?

Partly

Reviewer Expertise:

cataract surgery, statistical analysis

I confirm that I have read this submission and believe that I have an appropriate level of expertise to confirm that it is of an acceptable scientific standard, however I have significant reservations, as outlined above.

# Response

**Published:** 2022-12-26

**Authors:** Soujanya Kaup

**Affiliations:** Yenepoya (Deemed to be) University, India

1. Lack of controls to determine if the bevel-up vs. bevel-down technique was followed as per allocation.

**Response:** The surgeons are experienced in cataract surgeries and were familiar with both bevel-up and bevel-down phaco phacoemulsification. Immediately prior to starting the trial, the surgeons were asked to perform a minimum of 25 cases with each technique so that they adhere to the standard operating procedure for the surgical protocols in each of the trial arm.   At the end of each surgery, research assistant asks the surgeon to self-evaluate if they had any difficulty in adhering to the surgical protocol. If the surgeon states that they had to deviate from the surgical protocol, this would be noted and reported.   Also, a randomly selected sample of the surgical videos will be independently evaluated by a surgeon outside the trial and will be asked to assign them to bevel-up or -down groups and this will be compared to the original assignment. This is updated in the procotol.

 2. What percentage (time or energy) of the phacoemulsification must be done with the tip in this position. This aspect will probably affect the sample size calculation, as would the inclusion of confounding parameters such as centre/surgeon and grading strata.  

**Response: **The phaco-tip will be placed in the given position (based on the randomisation) throughout nucleus management (i.e., 100% of the time). Assuming the randomisation was effective in distributing cataract density between the two arms, this is not a concern – and we therefore don’t think it is necessary to use phaco time or energy as the driver for sample size calculation as the unit of randomisation is the patient not the “second of phaco time” or the “Watts of phaco power”.

 3. The follow-up time would probably not be long enough to see the final endothelial cell loss or decrease in central thickness. see for example DOI: 10.1007/s10792-016-0283-7    

**Response:** It can be acknowledged that the graph of CCT post phaco may not reach total stability until a later time point (60 or 90 days in the study quoted). However, we think it is reasonable to assume that the endothelial cell counts and CCT at earlier time points are extremely strong predictors for final outcomes. We see no reason to be concerned that bevel up, or bevel down, might cause a late drop in cell count 3 months after surgery compared to the other arm of the trial.   Also, studies have concluded that the rate of endothelial cell loss is maximum during the first month post-uneventful cataract surgery, and thereafter no accelerated cell loss takes place. Hence, we opted to choose post-op one month to evaluate endothelial count.  

- Beato JN, Esteves-Leandro J, Reis D, Falcão M, Rosas V, Carneiro Â, et al. Corneal structure and endothelial morphological changes after uneventful phacoemulsification in type 2 diabetic and nondiabetic patients. Arq Bras Oftalmol;84(5):454–61. Available from: http://www.ncbi.nlm.nih.gov/pubmed/34586219

4. I was unable to locate the data-set, maybe my fault.  

**Response:** There are no data-sets associated with this article. The DSMC Charter, Participant information sheet and Consent forms are available at https://doi.org/10.17605/OSF.IO/5YS6W

 5. How will the endothelial cell loss be recorded, absolute loss or percentage decrease, for the evaluation of between technique differences?

**Response: **Both ways of presenting the data are possible. Absolute number of endothelial cells lost will be measured in both the groups. Percentage of endothelial cell loss will also be calculated as = (preoperative cell count − postoperative cell count)/(preoperative cell count × 100%) Updated in the protocol.  

 6. SPSS 16 is a bit outdated. Consider using R?

**Response:** STATA 17 will be used. Updated in the protocol.
